# A robust method for approximate visual robot localization in feature-sparse sewer pipes

**DOI:** 10.3389/frobt.2023.1150508

**Published:** 2023-03-06

**Authors:** S. Edwards, R. Zhang, R. Worley, L. Mihaylova, J. Aitken, S. R. Anderson

**Affiliations:** Department of Automatic Control and Systems Engineering, University of Sheffield, Sheffield, United Kingdom

**Keywords:** robot localization, sewer pipe networks, feature-sparse, visual odometry, bag-of-keypoints, pipe joint detection

## Abstract

Buried sewer pipe networks present many challenges for robot localization systems, which require non-standard solutions due to the unique nature of these environments: they cannot receive signals from global positioning systems (GPS) and can also lack visual features necessary for standard visual odometry algorithms. In this paper, we exploit the fact that pipe joints are equally spaced and develop a robot localization method based on pipe joint detection that operates in one degree-of-freedom along the pipe length. Pipe joints are detected in visual images from an on-board forward facing (electro-optical) camera using a bag-of-keypoints visual categorization algorithm, which is trained offline by unsupervised learning from images of sewer pipe joints. We augment the pipe joint detection algorithm with drift correction using vision-based manhole recognition. We evaluated the approach using real-world data recorded from three sewer pipes (of lengths 30, 50 and 90 m) and benchmarked against a standard method for visual odometry (ORB-SLAM3), which demonstrated that our proposed method operates more robustly and accurately in these feature-sparse pipes: ORB-SLAM3 completely failed on one tested pipe due to a lack of visual features and gave a mean absolute error in localization of approximately 12%–20% on the other pipes (and regularly lost track of features, having to re-initialize multiple times), whilst our method worked successfully on all tested pipes and gave a mean absolute error in localization of approximately 2%–4%. In summary, our results highlight an important trade-off between modern visual odometry algorithms that have potentially high precision and estimate full six degree-of-freedom pose but are potentially fragile in feature sparse pipes, *versus* simpler, approximate localization methods that operate in one degree-of-freedom along the pipe length that are more robust and can lead to substantial improvements in accuracy.

## 1 Introduction

Sewer networks transport waste products in buried pipes. They are an essential part of our infrastructure but are prone to damage such as cracks, with an estimated 900 billion gallons of untreated sewage discharged into United States of America waterways each year (American Society of Civil Engineers, 2011). Therefore, sewer pipes need regular monitoring and inspection so that repairs can be effectively targeted and performed. The traditional way of performing inspection in sewer pipes is *via* manually operated, tethered, CCTV rovers. There is an opportunity to make this process more efficient *via* autonomous robot inspection. One of the key challenges to overcome for this is to solve the robot localization problem so that the location of damage is known.

There are a number of different methods developed for robot localization in pipes (Aitken et al., 2021; Kazeminasab et al., 2021). The methods can be divided based on sensor type: the most simple are dead-reckoning methods based on inertial measurement units (IMUs) and wheel or tether odometry (Murtra and Mirats Tur, 2013; Chen et al., 2019; Al-Masri et al., 2020). The main limitation of these methods is that they drift, and so some authors have introduced drift correction methods based on known landmarks including pipe joints (where accelerometers are used to detect the vibration as the robot moves over the joint) (Sahli and El-Sheimy, 2016; Guan et al., 2018; Wu et al., 2019; Al-Masri et al., 2020), and above-ground reference stations (Wu et al., 2015; Chowdhury and Abdel-Hafez, 2016).

Cameras are another widely-used method of localization in pipe robots, using monocular visual odometry (VO) (Hansen et al., 2011a; Hansen et al., 2013; Hansen et al., 2015), visual simultaneous localization and mapping (vSLAM) (Evans et al., 2021; Zhang et al., 2021), stereo VO (Hansen et al., 2011b), and RGB-D cameras (Alejo et al., 2017; Alejo et al., 2019). Laser scanners have been used in pipes for recognising landmarks such as manholes, junctions and elbows (Ahrary et al., 2006; Lee et al., 2016; Kim et al., 2018) although not, it would appear, for the odometry problem. Finally, acoustic and radio frequency (RF) signals such as ultrasonic (Ma et al., 2015), hydrophone (Ma et al., 2017a; Ma et al., 2017b; Worley et al., 2020a), RF (Seco et al., 2016; Rizzo et al., 2021), low frequency acoustic (Bando et al., 2016) and acoustic-echo (Worley et al., 2020b; Yu et al., 2023) methods have been used, but these are still emerging technologies.

The sensor technology that is most of interest in this paper for localization is cameras. This is because sewer pipe inspection is often conducted using vision-based methods (Duran et al., 2002; Myrans et al., 2018) and most pipe inspection robots developed to date include cameras for visual inspection, e.g., MAKRO (Rome et al., 1999), KANTARO (Nassiraei et al., 2006), MRINSPECT (Roh et al., 2008), PipeTron (Debenest et al., 2014), EXPLORER (Schempf et al., 2010) and recent miniaturized pipe inspection robots (Nguyen et al., 2022). Therefore, it is appealing to make dual use of a camera for both inspection and localization.

The main challenge facing camera-based localization in pipes is that standard visual odometry algorithms for localization based on keyframe optimisation methods, e.g., Hansen et al. (2011a); Hansen et al. (2013, 2015); Zhang et al. (2021); Evans et al. (2021) tend to fail in environments that lack visual features, and this is particularly the case for newer sewer pipes, although we have shown in aged sewer pipes that sufficient features exist for these methods to work well (Evans et al., 2021). We will go on to show in the results that a standard feature-based keyframe optimisation method for visual SLAM, ORB-SLAM3 (Campos et al., 2021), fails in these types of feature-sparse sewer pipe. There is a key research gap, therefore, in developing a visual odometry method for sewer pipes that lack visual features, which is the problem that we address here.

In this paper, we propose a new solution to the problem of robot localization in feature-sparse sewer pipes based on joint and manhole detections using camera images. Joint detection can be used for localization because joints occur at regularly spaced intervals where the inter-joint distance can be known *a priori* from installation data records, or estimated from odometry. The robot location along a pipe length, in one-degree of freedom, can be approximately obtained from scaling the count of pipe joints by the inter-joint distance. This transforms the problem of robot localization to one of pipe joint detection. Manholes can be mapped from above-ground to serve as drift-correcting landmarks when detected from inside the pipe to further improve the localization system.

Joint detection in pipes has been previously addressed using vision methods for the purpose of damage detection (not localization), using forward facing cameras (Pan et al., 1995), omni-directional cameras (Matsui et al., 2010; Mateos and Vincze, 2011) and fusion of laser scanners with cameras (Kolesnik and Baratoff, 2000). For forward facing cameras, the standard approach to pipe joint detection is to apply circle detection using the Hough transform to each image frame (Pan et al., 1995). However, in exploratory analysis we found the Hough transform approach was unreliable, often detecting spurious circles. Instead, here we use SURF for image feature extraction (Bay et al., 2008), followed by feature selection for pipe joints using a bag-of-keypoints method (Csurka et al., 2004), followed by circle fitting to detect the joint. The key advantage of our approach is that it enables us to train the feature selection algorithm on representative examples of sewer pipe joints (in an unsupervised manner), whilst the Hough transform does not have access to this prior information that specializes the method to the sewer pipe environment. We make our procedure even more robust by performing joint detections across a window of frames.

A potential limitation of only using pipe joints for localization is that detection errors can be made, such as a joint being missed (a false negative), or counted when not present (a false positive). To address this challenge, we develop a modular extension based on manhole detection to correct for drift in the localization algorithm: we assume the manhole locations are known or can be mapped from above-ground offline, and then use a linear classifier to detect the manholes from within the pipe. We use the *same* SURF image features as input to both the joint and manhole detection systems, making the approach more computationally efficient than using completely separate systems. Overall, the joint detection with manhole drift correction produces an accurate and robust method for localization.

**FIGURE 1:**
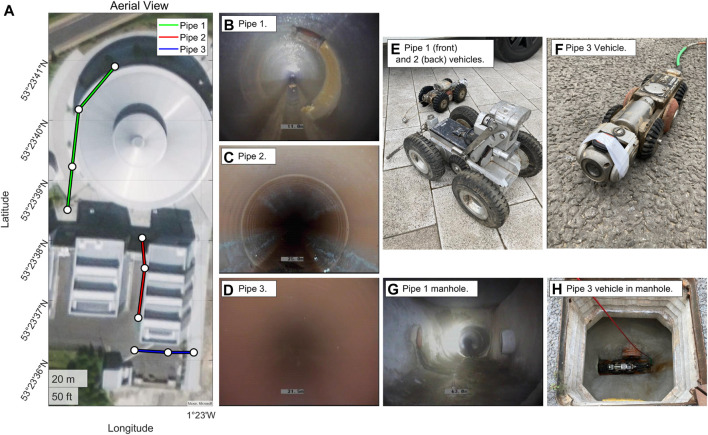
Sewer pipe environment and CCTV rovers used in testing and evaluation. **(A)** Aerial view of the three pipe used in testing (of diameters approximately Pipe 1: 600 mm, Pipe 2: 300 mm and Pipe 3: 150 mm). **(B–D)** Example images from inside the three pipes used in testing - note the general lack of visual features. **(E,F)** Example CCTV rovers used in testing. **(G)** Example manhole image. **(H)** Example manhole image viewed from above.

To test and evaluate the method, we use real sewer pipe data taken from three different types of pipe to demonstrate its effectiveness (Figure 1), data available at The University of Sheffield data repository ORDA https://figshare.shef.ac.uk/articles/dataset/Visual_Odometry_for_Robot_Localisation_in_Feature-Sparse_Sewer_Pipes_Using_Joint_and_Manhole_Detections_--_Data/21198070. We benchmark against ORB-SLAM3 (Campos et al., 2021) as a standard method for visual odometry.

In summary, the main contributions of the paper are as follows.

• A robust vision-based method for approximately localizing a robot (to the nearest pipe joint) along the lengths of feature-sparse sewer pipes using joint detections combined with a method for drift correction at manhole locations using vision-based automated manhole detection.
• A method for robustly detecting joints in pipes using a bag-of-keypoints visual categorization algorithm that is benchmarked against a standard method for detecting pipe joints in images - the Hough transform.
• Experimental testing and evaluation of the localization method using real-data gathered from three live sewer pipes.
• Benchmarking of the localization method against a well-known, state-of-the-art visual SLAM algorithm - ORB-SLAM3 (Campos et al., 2021).

The paper is structured as follows. In Section 2 we describe the methods and particularly our new algorithm for localization using pipe joint detections and manhole detections, as well as the dataset for evaluation. In Section 3 we give the results of the algorithm on real-world sewer pipe data and include a benchmark comparison to ORB-SLAM3. In Section 4 we provide a discussion and in Section 5 we summarise the main achievements of the paper.

## 2 Methods

**FIGURE 2:**
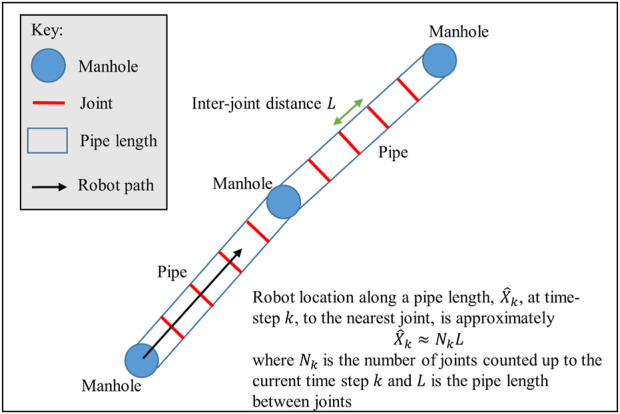
Diagram demonstrating how joint detection can be used to calculate the approximate robot location along the pipe length. The focus of this paper is on developing a robust vision-based method for detecting pipe joints from camera images to determine the joint count *N*_*k*_.

**FIGURE 3:**
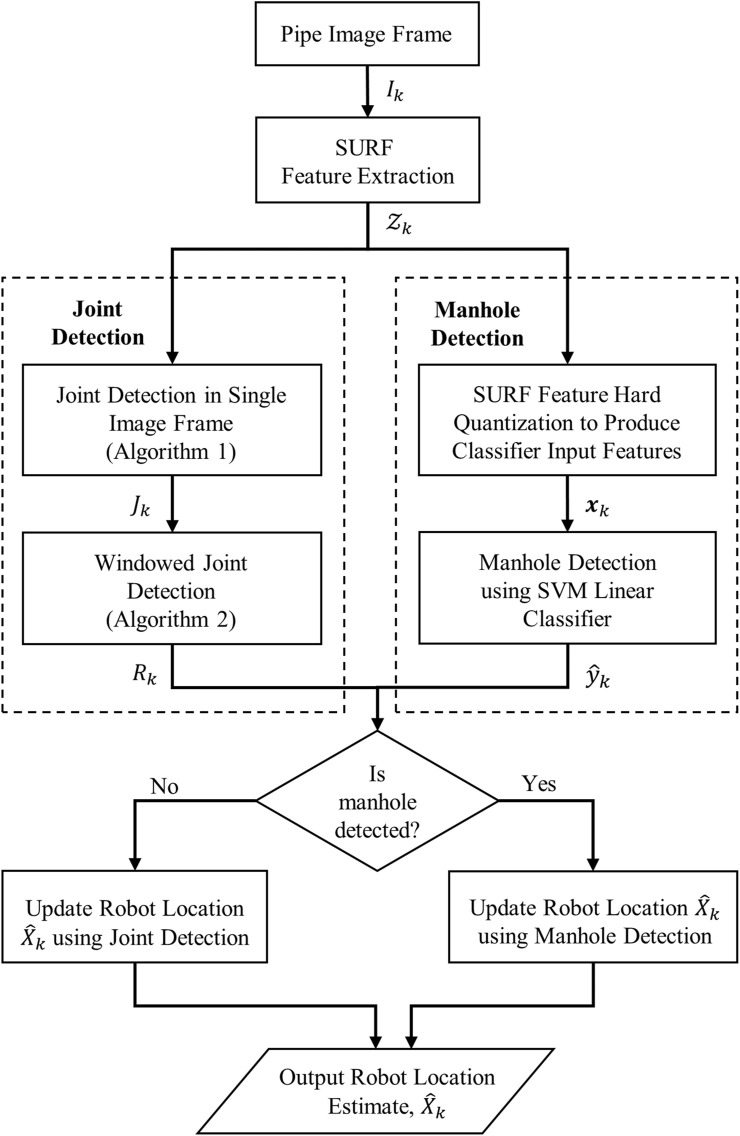
Overview of the robot localisation algorithm using joint and manhole detection. The pipe image *I*_*k*_ undergoes SURF features extraction to produce the features $Z_{k}$ . These features are transmitted to both the input of Algorithm 1 for joint detection and feature pre-processing in the Manhole detection pathway. Algorithm 1 for joint detection produces the flag *J*_*k*_ indicating presence or absence of a joint, which is the input to the windowed joint detection, described in Algorithm 2, which more robustly detects presence/absence of a joint from across a sliding window of individual joint detection. Both the output of windowed joint detection *R*_*k*_ and manhole detection ${\hat{y}}_{k}$ are analysed to check if a manhole is detected: if so then the robot location is updated using the manhole location and if not the robot location ${\hat{X}}_{k}$ is approximately updated using the most recent joint detection information, by calculating distance travelled from the joint count and inter-joint distance.

In this section we describe the joint detection algorithm, manhole detection and the experimental data collection used to evaluate the algorithm in a real-world live sewer pipe. The link between vision-based joint detection and robot localization along a pipe length is described in Figures 2, 3 gives an overview of the methods used for vision-based joint and manhole detection, and robot localization.

### 2.1 Joint detection

The robot location along the pipe, $\hat{X}$ , i.e., in one degree of freedom, can be obtained from joint detections because joints occur at regularly spaced intervals, where we assume the inter-joint distance is known *a priori* or we assume can be estimated. The joint detection algorithm proposed here has a few main steps described below: feature extraction, circle fitting and windowed joint detection.

The purpose of the feature extraction step in the algorithm is to find points of interest within the current test image frame *I*_*k*_ at time step *k*, to check for a joint. In this paper we use a method inspired by visual categorization with bags of keypoints (Csurka et al., 2004), because it is simple, fast and effective and therefore well suited to small, low-powered robots for the pipe environment. The method operates by first extracting features from a test image *I*_*k*_ using speeded up robust features (SURF) (Bay et al., 2008),

$$Z_{k}=\left\{z_{k,1},\ldots ,z_{k,n}\right\}$$ (1)

Where $z_{k,i}\in R^{d}$ is a SURF feature of dimension *d*, and *n* is the total number of SURF features found in an image. We then use a threshold test to retain only those features sufficiently similar to keypoints previously seen in joints in training data,

$$Z_{k}^{*}=\left\{z_{k,i}:d\left(z_{k,i},k_{j}\right)\leq \beta \right\}\;\forall i,j$$ (2)

Where *d*(**z**_*k*,*i*_, **k**_*j*_) is a distance-metric (Euclidean in this case) of feature **z**_*k*,*i*_ from keypoint **k**_*j*_ and *β* is a threshold parameter tuned offline. The keypoints, **k**_*j*_, are obtained offline by using a set of training data with a K-means clustering algorithm, similar to (Csurka et al., 2004). The use of multiple clusters enables the method to be robust to the variation in appearance of pipe joints, whilst also excluding non-joint features from detection. Identifying a joint does not require a large number of features, therefore it is more important to exclude false detections than to detect every relevant feature, so the threshold, *β* for selection is tuned to be more exclusive than inclusive. Additionally, points lying within regions of an image that are known to not contain joints, such as the centre, can be excluded automatically using a mask, *M*.

**FIGURE 4:**
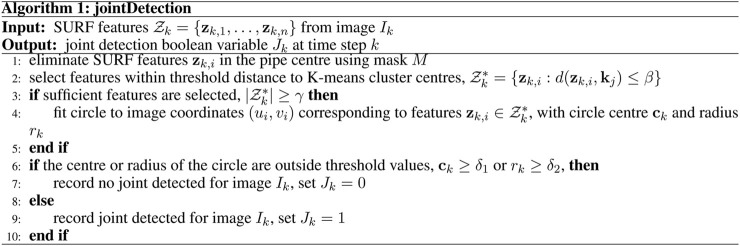
Algorithm 1: Joint detection in a single image frame.

We perform the actual joint detection by fitting a circle to the extracted features, $Z_{k}^{*}$ , to test whether the features resemble a joint. This is justified because it is known that sewer pipes are cylindrical, and the position of the robot’s camera is relatively central in the pipe during normal forward motion. As such, the pipe joints will appear close to circular in images captured by robots in pipes. Note that we only check for a joint if the number of features in $Z_{k}^{*}$ is greater than a threshold parameter, *γ*. Note also that the objective here is to detect the pipe joint, not obtain an optimal model of a circle, therefore we avoid computationally intensive circle fitting based on iterative optimisation (Gander et al., 1994), and opt for a more rapid and simple estimate: we take the average of all feature points as the circle centre, **c**_*k*_, and the average distance from the centre of each point as the radius, *r*_*k*_,

$$c_{k}=\left(\bar{u},\bar{v}\right)$$ (3)

$$r_{k}=\frac{1}{N}\overset{N}{\underset{i}{\sum }}\sqrt{{\left(u_{i}-\bar{u}\right)}^{2}+{\left(v_{i}-\bar{v}\right)}^{2}}$$ (4)

where (*u*_*i*_, *v*_*i*_) is the horizontal-vertical image coordinates corresponding to feature **z**_*k*,*i*_, $\bar{u}$ is the mean of the *u*-coordinates and $\bar{v}$ is the mean of the *v*-coordinates. This circle fitting method is computationally efficient but its disadvantage is that it will fit the circle to any set of points it is given, regardless of the actual geometry of the points (Gander et al., 1994). To compensate for this, the joint is only detected if the centre and radius of the fitted circle are within predefined threshold parameters *δ*_1_ and *δ*_2_ respectively. This procedure for detecting a joint in a single image frame *I*_*k*_ is described in Algorithm 1 (Figure 4).

**FIGURE 5:**
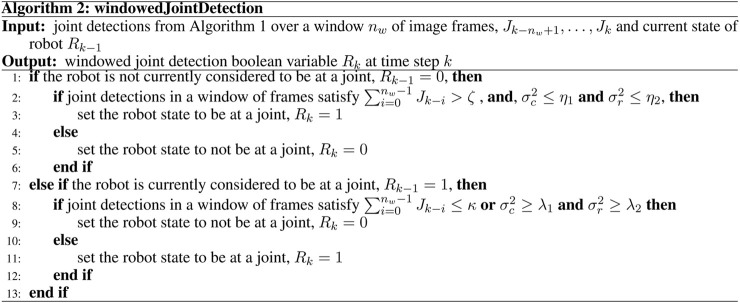
Algorithm 2: Detecting a joint in a window of frames.

The procedure for detecting a joint is made more robust here by performing detections across a window of frames. This reduces the impact of false detections, whilst also providing robustness against multiple correct, but discontinuous, detections of a single joint. To raise a joint detection flag, *R*_*k*_ = 1, the method simply requires that the number of positive joint detections from Algorithm 1, over a window of frames of size *n*_*w*_, is greater than a threshold parameter *ζ* and that the variances of the centre and radius of the detected joints are below thresholds, $\sigma_{c}^{2}\leq \eta_{1}$ and $\sigma_{r}^{2}\leq \eta_{2}$ . The reverse procedure is used to lower the joint detection flag, *R*_*k*_ = 0. This procedure is described in Algorithm 2 (Figure 5).

All parameters in Algorithms 1 and 2 were tuned using a grid search across real sewer pipe data (except *γ*, *δ*_1_, *δ*_2_ and *ζ* that were tuned manually). The grid search took a number of days to evaluate because it involved a relatively long distance with a large number of parameters (*β*, *η*_1_, *η*_2_, *n*_*w*_, *λ*_1_, *λ*_2_) and although reaching a global optimum could not be guaranteed, as a global search method a grid search is relatively robust to not well-distinguishable local optima, and testing on independent validation data ensured good generalization.

The robot location ${\hat{X}}_{k}$ along a pipe, can be obtained relative to a starting position by using the joint detections, *R*_*k*_, to count the number of joints detected up to the current time step, *N*_*k*_, and then taking the product of the joint count with the inter-joint distance, *L*,

$${\hat{X}}_{k}\approx N_{k}L.$$ (5)

### 2.2 Manhole detection

Manhole detections can be used to correct drift in the joint localization algorithm. Manhole locations can be known *a priori* or mapped from above-ground, and then detected from within the pipe. While many potential methods of detecting manholes exist, we continue to rely on camera data and use a bag-of-features image recognition system to detect manholes. The advantage of this approach is that the *same* SURF features extracted for joint detections, $Z_{k}$ , can be used for manhole detection. This makes the approach more computationally efficient than using a separate feature extraction/classification method.

To detect manholes, we define a standard binary classification problem of manhole *versus* no manhole, using a linear support vector machine (SVM). We construct a visual vocabularly of features offline along with clusters using K-means clustering (Wang and Huang, 2015), and then in online operation the SURF features, **z**_**k**_, extracted from image frame *I*_*k*_, undergo hard quantization by representing each local feature by the nearest visual word, which produces the classifier input features **x**_*k*_. The classifier training/validation dataset of input-output pairs is therefore represented as

$$D=\left\{\left(x_{1},y_{1}\right),\ldots ,\left(x_{m},y_{m}\right)\right\},$$ (6)

where the binary output *y*_*k*_ ∈ { + 1, −1}, represents the classes manhole and no manhole respectively. We use a standard linear (soft margin) SVM to classify the presence of a manhole, where the decision hyperplane is defined as

$$f\left(x_{k}\right)=w^{T}x_{k}+b=0,$$ (7)

where **w** and *b* are the parameters that define the decision hyperplane and the classifier predicts the class label using the sign of *f*(**x**_*k*_). Optimal parameters for the soft-margin SVM were estimated here using sequential minimal optimization (Cervantes et al., 2020) using a balanced dataset of sample images taken from pipes 1, 2 and 3, and performance was evaluated using 10-fold cross-validation.

In online operation detections of manholes are windowed similarly to joints (as in Figure 3) in order to limit the impact of single errant frames. Finally, the robot location, ${\hat{X}}_{k}$ , can be determined from the any detected manhole because manhole locations are assumed known.

### 2.3 Experimental data

Tests were conducted on data gathered from real-world, live, buried sewer pipes at The Integrated Civil and Infrastructure Research Centre (iCAIR), at The University of Sheffield, UK, using tethered mobile CCTV platforms commonly used in pipe inspections. As can be see in Figure 1, three different platforms were used to allow a variety of pipes to be tested.

The pipe lengths were approximately 90 m for pipe 1, 50 m for pipe 2 and 30 m for pipe 3. Diameters were approximately 600 mm for pipe 1, 300 mm for pipe 2 and 150 mm for pipe 3.

The data used to train both the joint detection and manhole detection systems was selected in part from the data used to test the systems, but also from data in other pipes at the same location and collected in the same experiment.

Ground truth localization was obtained from the robot tether, which measured distance travelled.

### 2.4 Testing and evaluation

The localization system proposed here was compared and benchmarked against a standard method for visual odometry, ORB-SLAM3. To do this, the camera intrinsics were obtained from a standard checkerboard calibration procedure.

To evaluate the overall effectiveness of the robot localization system using our proposed method and ORB-SLAM3 we used the mean absolute error in localization,

$$\text{MAE}=\frac{1}{n}\overset{n}{\underset{i=1}{\sum }}|X_{i}-{\hat{X}}_{i}|,$$ (8)

where *X*_*i*_ is the true robot location in terms of distance travelled along the pipe map (as measured using the tether on the robot) and ${\hat{X}}_{i}$ is the estimated robot location.

We also used the percent of operating time spent with an error below a threshold,

$$E_{\%}=\frac{100}{n}\overset{n}{\underset{i=1}{\sum }}g\left(X_{i}\right),$$ (9)

where

$$g\left(X_{i},{\hat{X}}_{i}\right)=\left\{\begin{array}{cc} 1 & |X_{i}-{\hat{X}}_{i}|\leq E_{\text{thresh}}\\ 0 & |X_{i}-{\hat{X}}_{i}|>E_{\text{thresh}} \end{array}\right.$$ (10)

and where *E*_thresh_ is the known distance between each pipe joint, which would be the maximum error if the system was performing ideally.

To evaluate the manhole classifier and joint detections we used the metrics accuracy *A* (manholes only), recall *R*, precision *P* and F1 score *F*_1_ (Powers, 2011).

$$A=\frac{\text{TP}+\text{TN}}{\text{TP}+\text{TN}+\text{FP}+\text{FN}},$$ (11)

$$P=\frac{\text{TP}}{\text{TP}+\text{FP}},$$ (12)

$$R=\frac{\text{TP}}{\text{TP}+\text{FN}},$$ (13)

$$F_{1}=2\frac{P.R}{P+R},$$ (14)

Where TP is true positive, TN is true negatives, FP is false positives and FN is false negatives.

## 3 Results

### 3.1 Feature detection in sewer pipes vs urban environments

**FIGURE 6:**
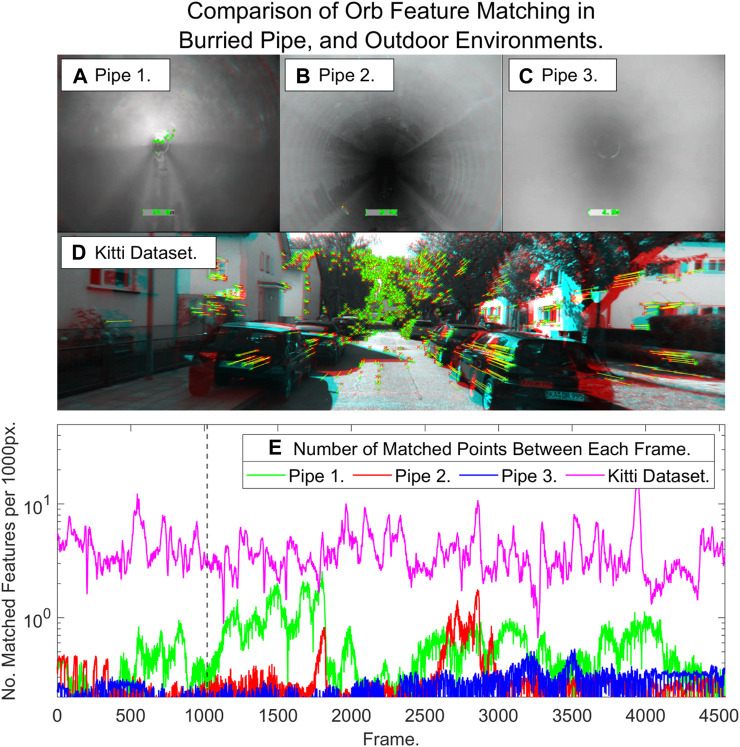
ORB feature extraction and matching in **(A–C)** sewer pipes *versus* **(D)** and outdoor scene from the KITTI dataset (matched points are shown in red and green pairs connected by yellow lines). **(E)** Quantification of feature matching normalised by 1000 pixels (note the logarithmic scale).

In this section we analyse the nature of the sewer pipe environment with regard to the prevalence of features and compare to outdoor urban environments where visual odometry is often applied. We compare ORB feature extraction and matching in our sewer pipes to a sequence from the well-known KITTI dataset (Geiger et al., 2013): as can be seen in Figure 6A–C *versus* Figure 6D, images from pipe interiors often contain significantly less regions of high texture than those in outdoor environments where visual SLAM systems are known to work well. Figure 6E shows that the number of features matched often an order of magnitude lower than in an outdoor environment. This causes frame by frame feature matching algorithms to frequently fail in sewer pipes.

### 3.2 Joint detection

**FIGURE 7:**
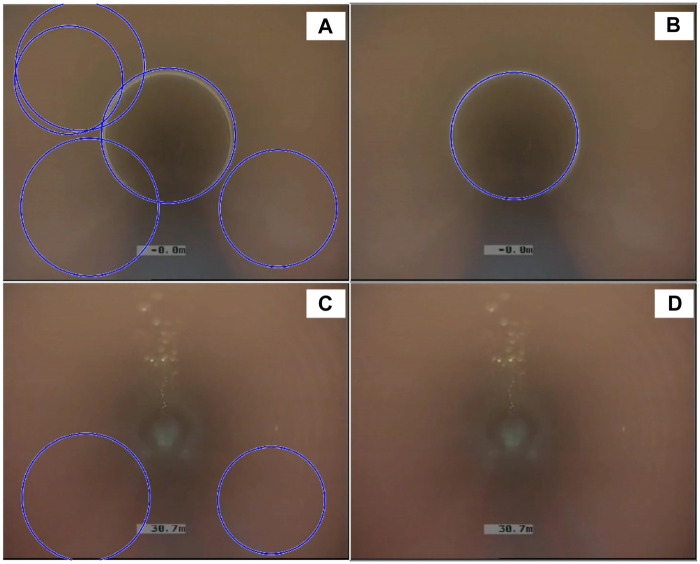
Pipe joint detection using our proposed method based on a bag-of-keypoints feature recognition method *versus* the Hough transform. **(A)** Hough transform: the single joint is correctly detected but there are also multiple false positive circle detections (with different radii). **(B)** Bag-of-keypoints method: the single joint is correctly detected with no false positives. **(C)** Hough transform: there is no joint present in the frame but the Hough transform still detects circles. **(D)** Bag-of-keypoints method: no joint is present in the frame, which the method correctly detects.

In this section, we provide the results of our proposed joint detection algorithm (Algorithm 1 only, i.e., no windowing) along with a comparison to the Hough transform, which is a standard method of circle detection in images (Yuen et al., 1990), as used in the Matlab function *imfindcircles* and the OpenCV function *cv2. HoughCircles*. To evaluate and compare the methods we used all image frames from pipe 2 and varied the Hough transform tuning parameters, edge threshold and sensitivity, systematically to search out the best performance. The results demonstrate the problems with using the Hough transform compared to our method (Table 1) - the edge threshold parameter requires tuning to a value of zero to detect any circles, which leads to a large number of false positives. Consequently, the accuracy is generally low, approximately 25% using the Hough transform (with edge threshold zero and sensitivity 0.9), mainly due to the detection of large numbers of false positives, compared to 85%, using our proposed method of joint detection (Figure 7A–D).

**TABLE 1:** Comparison of joint detection using the bag-of-keypoints method *versus* the Hough transform (where the edge threshold and sensitivity of the Hough transform are varied systematically). Note that in certain instances the Hough transform fails to detect any circles therefore Precision and F1-score are undefined, which is indicted with a “-”.

	Joint detection with bag-of-keypoints	Hough transform							
Edge Thresh	-	0	0.25	0	0.25	0	0.25	0	0.25
Sensitivity	-	0.85	0.85	0.9	0.9	0.95	0.95	1	1
Accuracy	84.65%	57.35%	58.01%	24.67%	58.01%	5.38%	58.01%	5.77%	0.66%
Precision	0.85	0.00	-	0.08	-	0.05	-	0.06	0.01
Recall	0.73	0.00	0.00	0.16	0.00	0.14	0.00	0.16	0.02
F1-score	0.78	-	-	0.11	-	0.08	-	0.08	0.01

### 3.3 Manhole detection

**FIGURE 8:**
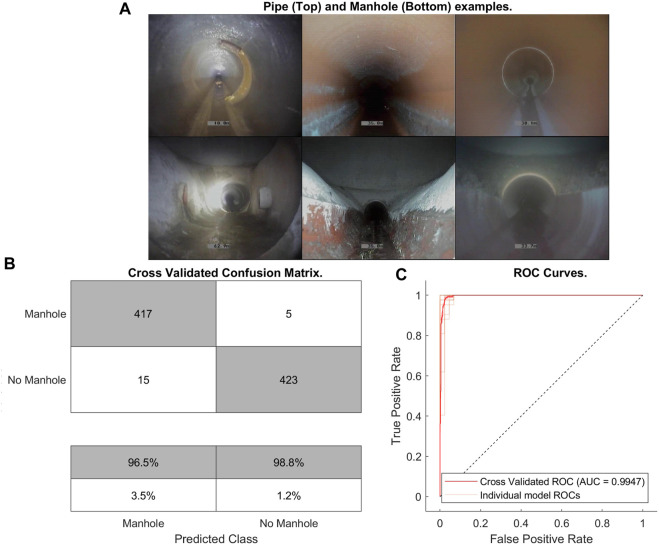
Manhole detection results. **(A)** Example images from pipes 1, 2 and 3 (left to right) respectively of the pipe image (top) *versus* the manhole image (bottom). **(B)** Classification confusion matrix from cross-validation data. **(C)** ROC curve for manhole detection.

In this section we provide results of manhole detections using using linear SVM classification. Manhole detection performed well, with an accuracy on 10-fold cross validation of 98.5% (with precision 0.97, recall 0.99 and F1-score 0.98): Figure 8A shows examples of pipe and manhole environments which illustrates the high visual difference between the two, as well as the spatial distinctions which my be exploitable by other sensors. 10-fold cross validation was performed on a selection of images from pipes and manholes across a variety of pipes.

The network displays a high accuracy as can be seen in Figure 8B as well as in Figure 8C which also indicates both a high specificity and sensitivity. The shown accuracy is sufficient for the system to reliably detect every manhole it encounters, and a windowing method similar to that used in the joint detection algorithm prevents any false positives or negatives from causing the same manhole to be detected twice.

### 3.4 Localization

**FIGURE 9:**
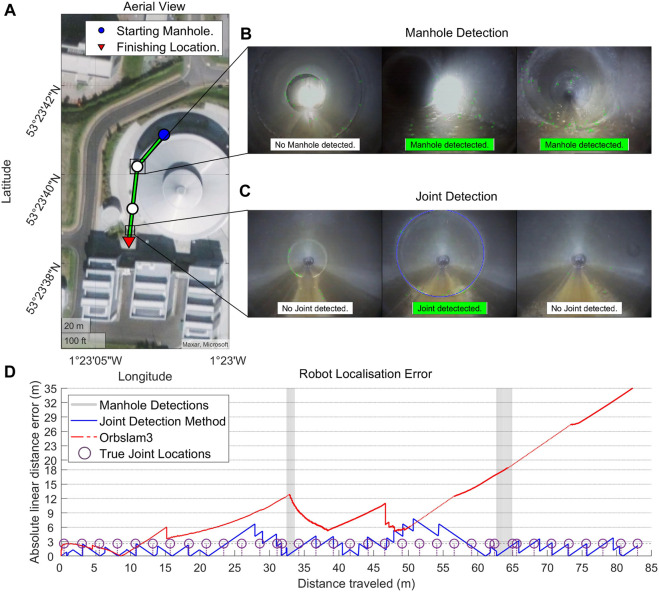
Localization results in Pipe 1. **(A)** Aerial view of pipe 1 with manhole locations highlighted as circles. **(B)** Example in-pipe view of a manhole, with successful manhole detection highlighted in green. **(C)** Example in-pipe view of a pipe joint, with successful joint detection highlighted in green. **(D)** Comparison of errors in localization using the method of joint detection with manhole correction versus ORB-SLAM3, with loss of tracking in ORB-SLAM3 denoted as a dashed red line.

Localization accuracy using the proposed joint detection algorithm improved substantially over using ORB-SLAM3: the average mean absolute error for the joint detection algorithm was 1.8 metres, whilst for ORB-SLAM3 it was 11.5 m in pipes 1 and 2 and a complete failure in pipe 3 due to lack of features. Joint detection, however, worked well in all pipes tested with an F1-score of 0.72–0.95 (Table 2). It is worth also emphasising that although ORB-SLAM3 produced a result in pipes 1 and 2, it still frequently lost feature tracking and re-initialised due to lack of feature matching. Figure 9 provides a detailed illustration and comparison of localization methods in pipe 1.

**TABLE 2:** Localization results from pipe joint detection *versus* ORB-SLAM3 from three sewer pipes.

	Joint detection results			Localization accuracy					
Pipe	Recall	Precision	F1 Score	Mean Absolute Error (m)			Time Spent Under Target Error (%)		
				Joint + Manhole	Joint only	ORB-SLAM3	Joint + Manhole	Joint only	ORB-SLAM3
Pipe 1	0.69	0.76	0.72	2.04	2.25	12.70	70.78	69.15	11.52
Pipe 2	1	0.80	0.89	2.11	4.23	10.36	72.90	37.30	15.38
Pipe 3	1	0.91	0.95	1.12	1.03	N/A	97.53	97.53	N/A

Table 2 also shows measured error metrics from the Joint Odometry, both with and without manhole correction, and compares them to ORB-SLAM3. The localization results using ORB-SLAM3 correspond to a mean absolute error of approximately 12%–20% on pipes 1 and 2 (failure in pipe 3), whilst our method worked successfully on all tested pipes and gave a mean absolute error in localization of approximately 2%–4% across all pipes, which was a substantial improvement.

## 4 Discussion

### 4.1 Overall performance

The aim of this paper was to develop a localization system for feature-sparse sewer pipes based on visual joint and manhole detection, to overcome the limitations of conventional keyframe optimisation visual odometry systems. The results have demonstrated that this objective was successfully achieved. While the system lacks the precision often desired for odometry systems due to its discrete nature, it is able to perform highly accurate localization given relatively limited knowledge of the operating environment. Additionally, discrete updates based on prior external information free the system from problems such as scale ambiguity and loss of tracking that are particularly difficult to overcome in pipe environments. Finally, while still present in the system, errors are accumulated every distance update rather than every frame and are smaller relative to the update than in traditional visual odometry systems, meaning that drift cannot accumulate quick enough to cause system failure before manhole detection corrects the state estimate.

The systems main cause of failure is lower precision and recall in more varied pipe environments, however it should be noted that the systems parameters can be optimised to improve performance in a single pipe at the expense of others.

### 4.2 Future work

In future work, a number of improvements could be investigated to the system presented here. The first improvement would be an online adaptive method for adjusting the joint detection algorithm parameters automatically while in operation, to account for minor differences between pipes.

The second improvement would be to automatically estimate inter-joint distances. Here we assume these distances are known *a priori*, which is realistic for some pipes. However, this knowledge might not always be available. We have found that simple odometry methods, such as wheel odometry, may be accurate enough over the short distances between pipe joints to derive this information during operation. Alternatively, it should be possible to use the detection of manholes, mapped from above-ground, to estimate the inter-joint distances. These methods require development and testing in future work. In addition, future work could address specific problems where joints are irregularly spaced and pipe bends occur - the latter problem could be addressed by combining joint detection for measuring distance travelled with an IMU to sense changes of direction.

Thirdly, false positives are primarily associated with manholes, however, other predictable environmental features, such as connecting pipes, are also known to reduce the accuracy of the detection system. These other predictable features provide opportunity for further improvement through their detection or further exploitation of prior knowledge.

## 5 Summary

In this paper we developed a localization method for sewer pipe inspection robots, operating in pipes with sparse visual features. The method exploited the intrinsic characteristic of the sewer pipe environment, that pipe joints occur at regularly spaced intervals. Therefore the localization problem was transformed to one of pipe joint detection. To further robustify the procedure, manhole detection was also included, which enabled drift correction based on manholes that could be mapped from above-ground. The visual localization algorithm was evaluated on three different real-world, live sewer pipes and then benchmarked against a standard method for visual odometry - ORB-SLAM3. We showed that our method substantially improved on the accuracy and robustness of ORB-SLAM3. Whilst visual SLAM algorithms such as ORB-SLAM3 are sophisticated, potentially very accurate and estimate the full six degree-of-freedom robot pose compared to our discrete, approximate localization method that only works in one-dimension along the pipe length, we would note that there is a trade-off in accuracy and robustness here, with ORB-SLAM3 regularly failing to track features in these feature-sparse sewer pipes. Ultimately we might find both systems are used in parallel in future to take advantage of the attributes of both approaches. The developed method can be applied as part of real robot localization systems, as part of digital twins for pipe networks with different scale, under different environmental conditions and different prior knowledge.

## Data Availability

The datasets presented in this study can be found in online repositories. The names of the repository/repositories and accession number(s) can be found below: https://figshare.shef.ac.uk/articles/dataset/Visual_Odometry_for_Robot_Localisation_in_Feature-Sparse_Sewer_Pipes_Using_Joint_and_Manhole_Detections_--_Data/21198070.
